# Optimized workflow to modify microRNA expression in primary human intravascular cells

**DOI:** 10.1186/s12865-023-00540-9

**Published:** 2023-02-15

**Authors:** Safak Caglayan, John-Bjarne Hansen, Omri Snir

**Affiliations:** 1 Thrombosis Research Center (TREC), Institute of Clinical Medicine, UiT – The Arctic University of Norway, Tromsø, Norway; 2 Division of Internal Medicine, University Hospital of North Norway, Tromsø, Norway

**Keywords:** miRNA inhibitor, miRNA mimic, Endothelial cell, Monocyte, Transfection, Gymnosis

## Abstract

**Background:**

A comprehensive dissection of the role of microRNAs (miRNAs) in gene regulation and subsequent cell functions requires a specific and efficient knockdown or overexpression of the miRNA of interest; these are achieved by transfecting the cell of interest with a miRNA inhibitor or a miRNA mimic, respectively. Inhibitors and mimics of miRNAs with a unique chemistry and/or structural modifications are available commercially and require different transfection conditions. Here, we aimed to investigate how various conditions affect the transfection efficacy of two miRNAs with high and low endogenous expression, miR-15a-5p and miR-20b-5p respectively, in human primary cells.

**Results:**

MiRNA inhibitors and mimics from two commonly used commercial vendors were employed, i.e., mirVana (Thermo Fisher Scientific) and locked nucleic acid (LNA) miRNA (Qiagen). We systematically examined and optimized the transfection conditions of such miRNA inhibitors and mimics to primary endothelial cells and monocytes using either a lipid-based carrier (lipofectamine) for delivery or an unassisted uptake. Transfection of LNA inhibitors with either phosphodiester (PE)- or phosphorothioate (PS)-modified nucleotide bonds, delivered using a lipid-based carrier, efficiently downregulated the expression levels of miR-15a-5p already 24 h following transfection. MirVana miR-15a-5p inhibitor displayed a less efficient inhibitory effect, which was not improved 48 h following a single transfection or two consecutive transfections. Interestingly, LNA-PS miR-15a-5p inhibitor efficiently reduced the levels of miR-15a-5p when delivered without a lipid-based carrier in both ECs and monocytes. When using a carrier, mirVana and LNA miR-15a-5p and miR-20b-5p mimics showed similar efficiency 48 h following transfection to ECs and monocytes. None of the miRNA mimics effectively induced overexpression of the respective miRNA when given to primary cells without a carrier.

**Conclusion:**

LNA miRNA inhibitors efficiently downregulated the cellular expression of miRNA, such as miR-15a-5p. Furthermore, our findings suggest that LNA-PS miRNA inhibitors can be delivered in the absence of a lipid-based carrier, whereas miRNA mimics need the aid of a lipid-based carrier to achieve sufficient cellular uptake.

**Supplementary Information:**

The online version contains supplementary material available at 10.1186/s12865-023-00540-9.

## Background

MiRNAs are small, non-coding RNAs that are critical for post-transcriptional regulation of gene expression. MiRNAs have preferential conserved interaction with most human mRNAs, and thereby significantly contribute to almost every developmental and physiological process [1]. Supporting this notion, dysregulation of miRNA biogenesis and/or function is associated with various diseases including immune system disorders [2], cardiovascular diseases [3], cancer [4], and neurodegenerative disorders [5]. As dysregulation of miRNAs is found to be casual in various diseases and miRNAs can fine-tune various pathways simultaneously, targeting of miRNAs has emerged as a promising therapeutic tool [6]. Specific targeting of miRNAs, which is used to downregulate their expression and activity, is achieved using synthetic miRNA inhibitors. These are antisense oligonucleotides designed to target endogenous miRNAs with high specificity by binding and sequestering their targets. MiRNA mimics are used to upregulate the expression of selected endogenous miRNAs. These are mostly small double-stranded oligonucleotides that match a corresponding endogenous miRNA sequence and are designed to simulate natural miRNAs in the cells.

The specificity and target regulation of miRNA inhibitors and mimics are determined by their nucleotide base sequence, primarily by the seed sequence at position 2–7 [1]. In addition to the sequence specificity, the delivery systems, and the potency of the oligonucleotides are important factors for the success of miRNA targeting and regulation. Importantly, unmodified oligonucleotides have limited efficacy due to rapid degradation and suboptimal delivery [7]. Therefore, lipid-based carriers are widely used to protect such oligonucleotides from degradation when delivered to cells. Lipid-based carriers are cationic lipid formulations that are commonly used as carriers due to their versatile use, effectiveness in encapsulation of oligonucleotides, and assistance in endosomal escape of the oligonucleotides [8]. However, toxicity of lipid-based carriers due to their charge and potential immunostimulatory effects are challenges to consider [9, 10]. As the potency of the oligonucleotides primarily depends on their chemistry, novel chemical and structural modifications were applied to maximize their effectiveness. Synthetic modifications in the phosphate group, nitrogenous bases, deoxyribose, and strand architecture are used [6, 7]. Phosphorothioate modification of the phosphate backbone is one of the most widely used chemical modifications that provide nuclease resistance [11]. Further changes can include locked nucleic acid (LNA), which links 2′-oxygen and 4′-carbon of ribose sugar and significantly increases binding affinity of the oligonucleotides. Chimeric oligonucleotides, which contain both LNA modified and unmodified ribose sugars, are also produced to decrease negative effects caused by certain modifications.

Downregulation or overexpression analysis of miRNAs in cells is critical in preclinical and functional studies [6]. There are currently several types of commercially available miRNA mimics and inhibitors, which harbor various patterns of chemical modifications. Chemical and structural alterations of some of these oligonucleotides are revealed to the users, while others contain modifications that are closed-source products. Endothelial cells (ECs) and intravascular cells such as monocytes are relevant targets in various conditions, such as cardiovascular diseases, thrombosis, and cardiac injury. Targeting of miRNAs was achieved in ECs or intravascular cells using various concentrations of miRNA inhibitors and mimics together with lipid-based carriers in previous studies [12–18]. However, a detailed workflow to inhibit or overexpress miRNAs with a systematic dose–response analysis of the synthetic miRNA inhibitors and mimics, and the importance of lipid-based carriers in delivery of such oligonucleotides to human primary cells have not been shown.

Here, we investigated the performance of commonly used and commercially available miRNA inhibitors and mimics, i.e., mirVana and Qiagen-LNA, with and without a lipid-based carrier in primary ECs and monocytes. We targeted miR-15a-5p, which is highly expressed, and miR-20b-5p that has low or undetectable expression in such cells. We showed that miRNA inhibitors with LNA modifications are superior to knock down miR-15a-5p expression in primary cells. Of note, while natural phosphodiester backbone is effective to knock down miR-15a-5p expression when used together with a lipid-based carrier, phosphorothioate backbone is required for efficient unassisted uptake of miRNA inhibitors by the primary cells. MirVana and Qiagen-LNA miRNA mimics, which were used for overexpression of miR-15a-5p and miR-20b-5p, showed similar potency in primary cells. We found that miRNA mimics are effective even in lower concentrations than commonly used, and a lipid-based carrier was necessary to deliver the miRNA mimics efficiently to the primary cells.

## Materials and methods

### Primary human endothelial cell culture

Pooled primary human umbilical vein ECs were purchased from Thermo Fisher Scientific and PromoCell. ECs at passage (P)2–3 were used in all experiments. ECs (5 × 10^5^) were thawed in endothelial cell growth media 2 (PromoCell, cat. no. C-22111) and directly seeded into a T75 flask. Growth media was replaced the following day with fresh media to eliminate DMSO.

### Isolation and culture of primary human monocyte

Blood from healthy individuals was collected from the antecubital vein into blood collection tubes containing sodium citrate. All study participants signed a written informed consent. Peripheral blood mononuclear cells (PBMCs) were separated from blood on a Ficoll-Paque (GE Healthcare) density gradient using LeucoSep separation tubes (Greiner), following centrifugation at 800 g for 15 min at room temperature without brake. Monocytes were negatively selected from PBMCs using Classical Monocyte Isolation Kit, LS Columns, and MidiMACS™ Separators (all from Miltenyi) according to the manufacturer’s instructions. Isolated monocytes were cultured in RPMI 1640, GlutaMAX™ Supplement (Thermo Fisher Scientific) media supplemented with 10% premium grade fetal bovine serum (P-FBS) with low endotoxin (Biowest) and 1:200 (volume/volume) penicillin–streptomycin solution (Sigma).

### Flow cytometry

PBMCs and isolated monocytes were stained with LIVE/DEAD Fixable Far-Red Dead Cell Stain Kit (Thermo Fisher Scientific), Brilliant Violet 421 anti-human CD45 (2D1), PE anti-human CD63 (H5C6), Alexa Fluor 647 anti-human CD14 (63D3), PE/Cyanine5 anti-human HLA-DR (L243), and PE/Cyanine7 anti-human CD162 (KPL-1) or their respective mouse IgG isotype control antibodies (all from BioLegend). ECs were stained with LIVE/DEAD Fixable Far-Red Dead Cell Stain Kit, and Brilliant Violet 605 anti-human CD31 (WM59) or the respective mouse IgG1 isotype control antibodies (BioLegend). Heat treated monocytes or ECs were prepared by exposure to 60 °C for 10 min and used to separate live cells and dead cells on the histograms. Staining was performed in flow cytometry buffer (PBS supplemented with 2% P-FBS and 0.5 mM EDTA) for 30 min at ambient temperature. Unstained cells and cells stained with single antibodies were prepared to set a suitable compensation matrix to correct for fluorescent spillover. After 2× washes with flow cytometry buffer, cells were analyzed using CytoFLEX Flow Cytometry instrument (Beckman Coulter). Data analysis was performed using FlowJo, version 10.

### Transfection and unassisted uptake of miRNA inhibitors and mimics

Prior to the transfection of miRNA inhibitors or mimics, subconfluent ECs were detached using Accutase (Thermo Fisher Scientific), collected and centrifuged at 220 g for 3 min. ECs were resuspended in fresh Endothelial Cell Growth Media 2 (PromoCell) and seeded in 24-well or 12-well plates at 6 × 10^4^ or 1.2 × 10^5^ cell per well, respectively. Isolated human monocytes (5 × 10^5^ cells per well) were cultured in 12-well plates in 1 mL RPMI 1640, GlutaMAX™ Supplement media supplemented with 10% P-FBS and 1:200 (volume/volume) penicillin–streptomycin solution (Sigma).

MirVana miRNA inhibitors and mimics were purchased from Thermo Fisher Scientific. Chemical modifications in mirVana miRNA inhibitors and mimics are proprietary information of Thermo Fisher Scientific and were not revealed by the vendor. MiRCURY LNA miRNA inhibitors and mimics were purchased from Qiagen. The performance of each miRNA inhibitor or mimic was compared to its respective unspecific scrambled control, which was purchased from the same supplier. MiRNA inhibitors and mimics were reconstituted in sterile nuclease-free water to prepare stock solutions of miRNA inhibitors at 50 µM and of miRNA mimics at 66.7 µM.

Working solutions at lower concentrations were freshly prepared by diluting the respective stock solutions in Opti-MEM Reduced Serum Media (Thermo Fisher Scientific). miRNA inhibitors or miRNA mimics were mixed with Opti-MEM media (50 µL) in a sterile tube. For transfection experiments, 1 µL or 2 µL (for a well of 24-well or 12-well plate, respectively) of Lipofectamine RNAiMAX (Thermo Fisher Scientific) reagent were added to Opti-MEM/miRNA inhibitor or Opti-MEM/miRNA mimic mix in a sterile tube. Prepared mixes were incubated for 20 min at room temperature to form oligonucleotide-lipid complexes before adding to ECs or monocytes. For unassisted uptake experiments, Opti-MEM and miRNA inhibitor or mimic were mixed and added to ECs or monocytes. To allow optimal unassisted uptake (i.e., in the absence of a lipid-based carrier), the cell culture media for ECs or monocytes was not replaced for the whole transfection period, 48 h. When ECs were transfected using Lipofectamine RNAiMAX, cell culture media was replaced following the first 24 h of transfection to eliminate the lipid reagents. When monocytes were transfected with miRNA mimics with the aid of Lipofectamine RNAiMAX, cells were collected next day by centrifugation at 350 g for 5 min to eliminate lipid reagent, resuspended in fresh RPMI 1640, GlutaMAX™ Supplement media supplemented with 10% P-FBS and cultured until harvesting.

### RNA isolation and qRT-PCR

Total RNA isolation was performed using miRNeasy Micro Kit (Qiagen) and cDNA was synthesized from 5 ng or 10 ng of total RNA using miRCURY LNA RT Kit (Qiagen) according to the manufacturer’s instructions. cDNA stock solution was diluted 1:60 (volume/volume) and 3 µl was used for each reaction in 96-well plates. MiRCURY LNA SYBR Green PCR Kit together with miRCURY LNA miRNA PCR Assays (Qiagen) were used in qRT-PCR experiments. The following PCR assays were used to detect the expression of miRNAs: miR-15a-5p, miR-103a-3p, miR-20b-5p. MiR-103a-3p was used as reference miRNA to normalize the miRNA expression among different conditions. While small nucleolar RNAs might also be considered as reference genes to normalize miRNA expression, they are longer compared to miRNAs and may have different processing mechanism. These features of small nucleolar RNAs may introduce bias in cDNA synthesis and expression analysis. Therefore, miR-103a-3p was chosen as the reference miRNA based on technical guidelines from Thermo Fisher Scientific (A technical guide to identifying miRNA normalizers using TaqMan Advanced miRNA Assays White Paper—Publication number COL313020916) and Qiagen (miRCURY LNA miRNA SYBR Green PCR Handbook—Publication number HB-2431-002 10/2019), and previously published studies [19, 20]. qRT-PCR experiments were performed using a LightCycler 96 System (Roche) using the following program: Preincubation (95 °C for 120 s) followed by 45 cycles of 2-step amplification (95 °C for 10 s and 56 °C for 60 s) and final melting step. qRT-PCR results were analyzed, and cycle quantification (Cq) values were determined using LightCycler 96 Software (Roche). Relative gene expression was calculated using the Delta-Delta-Cq method and shown as mean ± standard error of mean (SEM).

### Microscopy analysis of primary human ECs

Primary ECs were analyzed 48 h after transfection with miRNA mimics complexed with Lipofectamine RNAiMAX. Phase-contrast images of live ECs were taken using a 10× objective mounted on a ZEISS Primovert microscope equipped with a ZEISS Axiocam microscope camera. Images were processed using ZEISS Labscope imaging application. Scale bar represents 100 µM.

## Results

### Transfection of primary ECs with miRNA inhibitors

MiR-15a-5p and its family members are associated with different types of cancer and cardiovascular diseases, some of which involve ECs [21, 22]. MiR-15a-5p is abundantly expressed in ECs and was selected to study the knockdown efficiency of different commercially available miRNA inhibitors. The performance of three different miR-15a-5p inhibitors was examined: MirVana miR-15a-5p inhibitor from Thermo Fisher Scientific, a chemically modified single-stranded RNA molecule, and two additional miR-15a-5p inhibitors from Qiagen with LNA chemistry in their ribose sugars. One LNA miR-15a-5p inhibitor (Qiagen) had phosphodiester internucleotide bonds (hereafter LNA-PE), and the other had phosphorothioate (PS)-modified internucleotide bonds (hereafter LNA-PS).

**Fig. 1:**
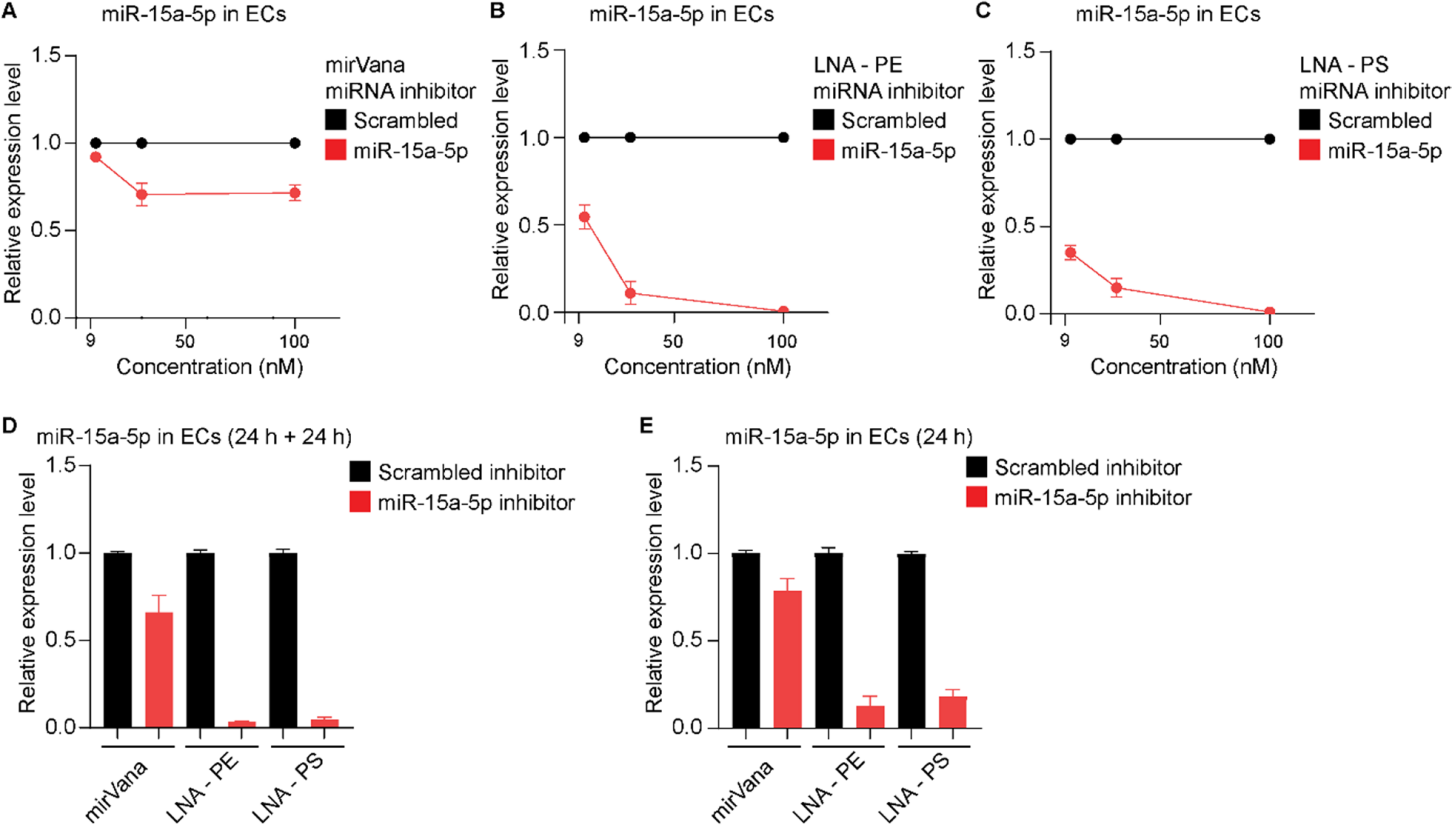
The effects of different miR-15a-5p inhibitors on the expression of miR-15a-5p in primary ECs. ECs were transfected with Lipofectamine RNAiMAX in complex with **A** mirVana, **B** LNA-PE or **C** LNA-PS miR-15a-5p inhibitors and their respective scrambled control at elevated concentration (9, 30 and 100 nM). Following 48 h, total RNAs were isolated and expression levels of miR-15a-5p and reference miRNA miR-103a-3p were measured by means of qRT-PCR. **D** MiR-15a-5p expression following two consecutive transfections, 24 h each, with 30 nM oligonucleotide-lipid complexes. **E** MiR-15a-5p expression in primary ECs following 24 h transfection using oligonucleotide-lipid complexes at 30 nM. Relative miR-15a-5p expression was shown as mean ± SEM. N = 3 experiments and N = 2 technical replicates were performed

First, we compared the performance of the three miR-15a-5p inhibitors when used in a complex with a lipid-based carrier, Lipofectamine RNAiMAX, which facilitates the delivery of oligonucleotides to cells. MiR-15a-5p inhibitors were pre-incubated with Lipofectamine RNAiMAX for 20 min at room temperature to attain oligonucleotide-lipid complexes. Next, elevated concentrations of the miR-15a-5p inhibitors (9 nM, 30 nM and 100 nM) in complex with Lipofectamine RNAiMAX were added to a culture of primary ECs. Total RNA samples were isolated from transfected cells 48 h after transfection and the efficiency of miR-15a-5p knockdown was evaluated using qRT-PCR. Figure 1A shows the effects of MirVana miR-15a-5p inhibitor on the expression levels of miR-15a-5p in ECs. While 9 nM had a minor effect on the expression of miR-15a-5p, 30 nM and 100 nM of the inhibitor reduced the expression of miR-15a-5p by approximately 30%. In comparison, both LNA-PE and LNA-PS miR-15a-5p inhibitors efficiently downregulated the expression of miR-15a-5p (Fig. 1B, C). Knockdown efficiency increased to > 90% when the LNA miRNA inhibitors were used at 30 nM, and a complete knockdown was achieved when used at 100 nM. LNA-PS miR-15a-5p inhibitor demonstrated the most potent inhibitory effect in comparison to the other two inhibitors when used at 9 nM (Fig. 1C).

Next, we tested whether two consecutive transfections within 48 h using miR-15a-5p inhibitors would further improve the efficacy. Complexes of 30 nM miR-15a-5p inhibitors and Lipofectamine RNAiMAX were added to EC culture. Following 24 h, a second dose of 30 nM of the respective miRNA inhibitor-lipid complexes were added to the cell culture. When mirVana miR-15a-5p inhibitor was used, the double-transfection protocol yielded an approximately 30% inhibition of endogenous miR-15a-5p, similar to that observed following single-transfection protocol. Double-transfection using the two LNA miRNA inhibitors resulted in > 99% downregulation of miR-15a-5p expression (Fig. 1D). The viability and cell surface expression of CD31 lineage marker in ECs was assessed following one-time and double transfection with the miRNA inhibitors. As shown in Additional file 1: Fig. S1, expression of CD31 and viability of ECs was not affected after a one-time transfection or a double transfection with mirVana, LNA-PE or LNA-PS miR-15a-5p inhibitors complexed with Lipofectamine RNAiMAX. Finally, we examined the effect of the different miR-15a-5p inhibitors following 24 h incubation. The two LNA miRNA inhibitors (30 nM) reduced the expression of miR-15a-5p in ECs by > 90% (Fig. 1E), whereas mirVana miR-15a-5p inhibitor (30 nM) showed a rather limited inhibitory effect, reducing the expression of miR-15a-5p by approximately 30% (Fig. 1E). Taken together, LNA-PE and LNA-PS miRNA inhibitors efficiently knocked down a highly expressed miRNA in primary ECs when delivered with the aid of a lipid-based carrier.

### Unassisted uptake of miRNA inhibitors by primary ECs

**Fig. 2:**
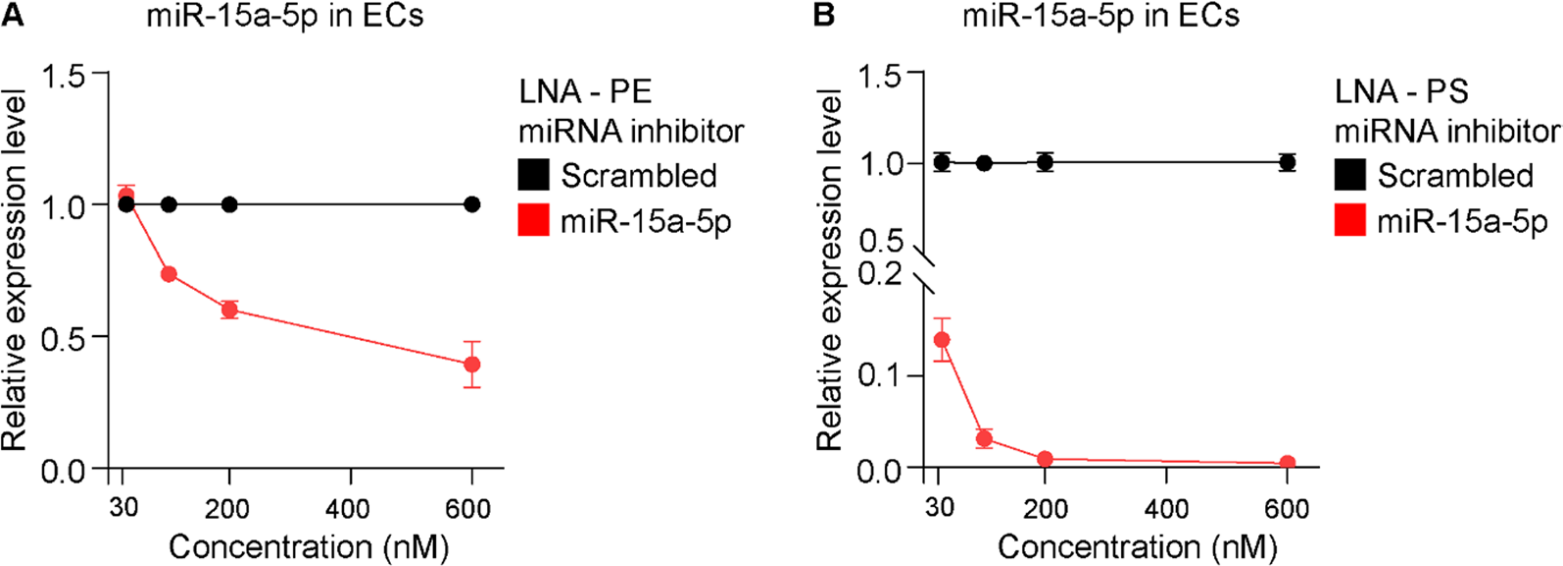
Expression of miR-15a-5p in ECs following unassisted uptake of LNA miR-15a-5p inhibitors. ECs were cultured with **A** LNA-PE or **B** LNA-PS miR-15a-5p inhibitors and their respective scrambled control at elevated concentration (30, 100, 200 and 600 nM). Following 48 h, total RNAs were isolated and expression levels of miR-15a-5p and reference miRNA miR-103a-3p were measured by means of qRT-PCR. Relative miR-15a-5p expression was shown as mean ± SEM. N = 2 experiments and N ≥ 2 technical replicates were performed

Oligonucleotides can be delivered directly to cells in the absence of a carrier, e.g., Lipofectamine RNAiMAX, in a process termed gymnosis. The efficient knockdown of miR-15a-5p by the two LNA miRNA inhibitors encouraged us to assess the efficiency of delivering LNA-PE and LNA-PS miR-15a-5p inhibitors to primary ECs in the absence of a lipid-based carrier. To this aim, LNA miRNA inhibitors or the respective scrambled control at elevated concentration were added directly to the culture media of primary ECs. Following 48 h of incubation, cells were harvested, total RNAs were extracted, and the expression of endogenous miR-15a-5p was analyzed using qRT-PCR. LNA-PE miR-15a-5p inhibitor, did not affect the expression of miR-15a-5p in primary ECs when used at 30 nM (Fig. 2A). Applying LNA-PE miR-15a-5p inhibitor at 100 nM, 200 nM and 600 nM directly to the cell culture resulted in 20%, 30% and 60% downregulation of miR-15a-5p expression, respectively (Fig. 2A). As shown in Fig. 2B, unassisted uptake of 30 nM LNA-PS miR-15a-5p inhibitor reduced the expression of miR-15a-5p by 85%. Higher concentration of the LNA-PS miR-15a-5p inhibitor reduced the expression level of miR-15a-5p by > 95%.

### Unassisted uptake of miRNA inhibitors by primary monocytes

**Fig. 3:**
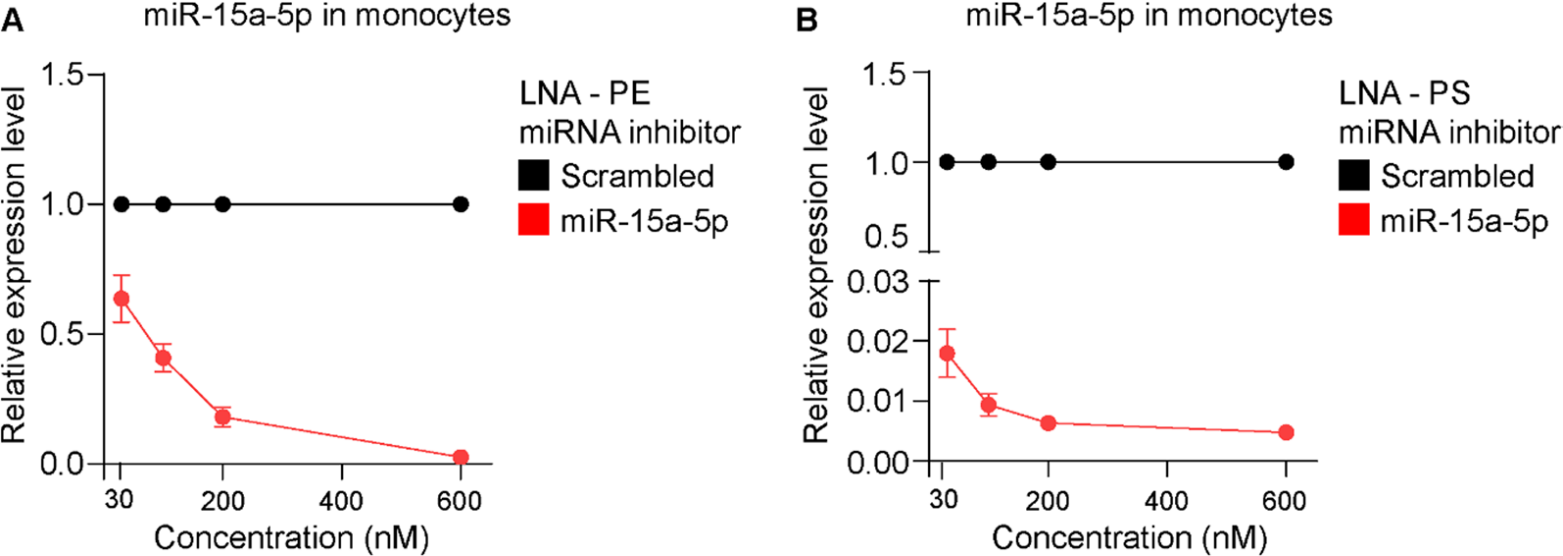
Expression of miR-15a-5p in primary monocytes following unassisted uptake of LNA mir-15a-5p inhibitors. Monocytes were cultured with **A** LNA-PE or **B** LNA-PS miR-15a-5p inhibitors and their respective scrambled control at elevated concentration (30, 100, 200 and 600 nM). Following 48 h, total RNAs were isolated and expression levels of miR-15a-5p and reference miRNA miR-103a-3p were measured by qRT-PCR. Relative miR-15a-5p expression was shown as mean ± SEM. N ≥ 3 experiments N ≥ 2 technical replicates were performed

Unassisted uptake of LNA miR-15a-5p inhibitors, and in particular LNA-PS, effectively downregulated the expression of miR-15a-5p in ECs. Next, we wished to assess the use of LNA miRNA inhibitors in primary human monocytes, used here to model non-proliferative, non-adherent primary cells. Freshly isolated monocytes were enriched from PBMCs, and their purity was determined by flow cytometry (Additional file 1: Fig. S2). Next, monocytes were incubated with LNA-PE or LNA-PS miR-15a-5p inhibitor, or their respective scrambled control. Following 48 h, the cells were harvested and the expression of endogenous miR-15a-5p was determined using qRT-PCR. Notably, the expression level of miR-15a-5p was reduced by approx. 98% when LNA-PS inhibitor was used at 30 nM (Fig. 3B). In comparison, higher concentrations of LNA-PE miR-15a-5p inhibitor were required to achieve similar downregulation of the endogenous miRNA (Fig. 3A). Taken together, these results suggest that LNA-PS miRNA inhibitor with chemically modified nucleotide bonds can be used without a lipid-based carrier to effectively knock down miRNA expression in human primary cells.

### Transfection of primary ECs with miRNA mimics

Next, we assessed the efficiency of lipid-based transfection and unassisted uptake of miRNA mimics to yield overexpression in primary cells. Mimic for miR-15a-5p, which is naturally expressed at high levels in primary ECs and monocytes was used, as well as a miRNA mimic for miR-20b-5p, which is expressed at low levels in these cells. MirVana and LNA miRNA mimics were tested as both are chemically modified double-stranded RNA molecules. As miRNA inhibitors effectively reduced the levels of the respective miRNA 48 h after transfection, this time point was also selected to evaluate the effects of miRNA mimics.

**Fig. 4:**
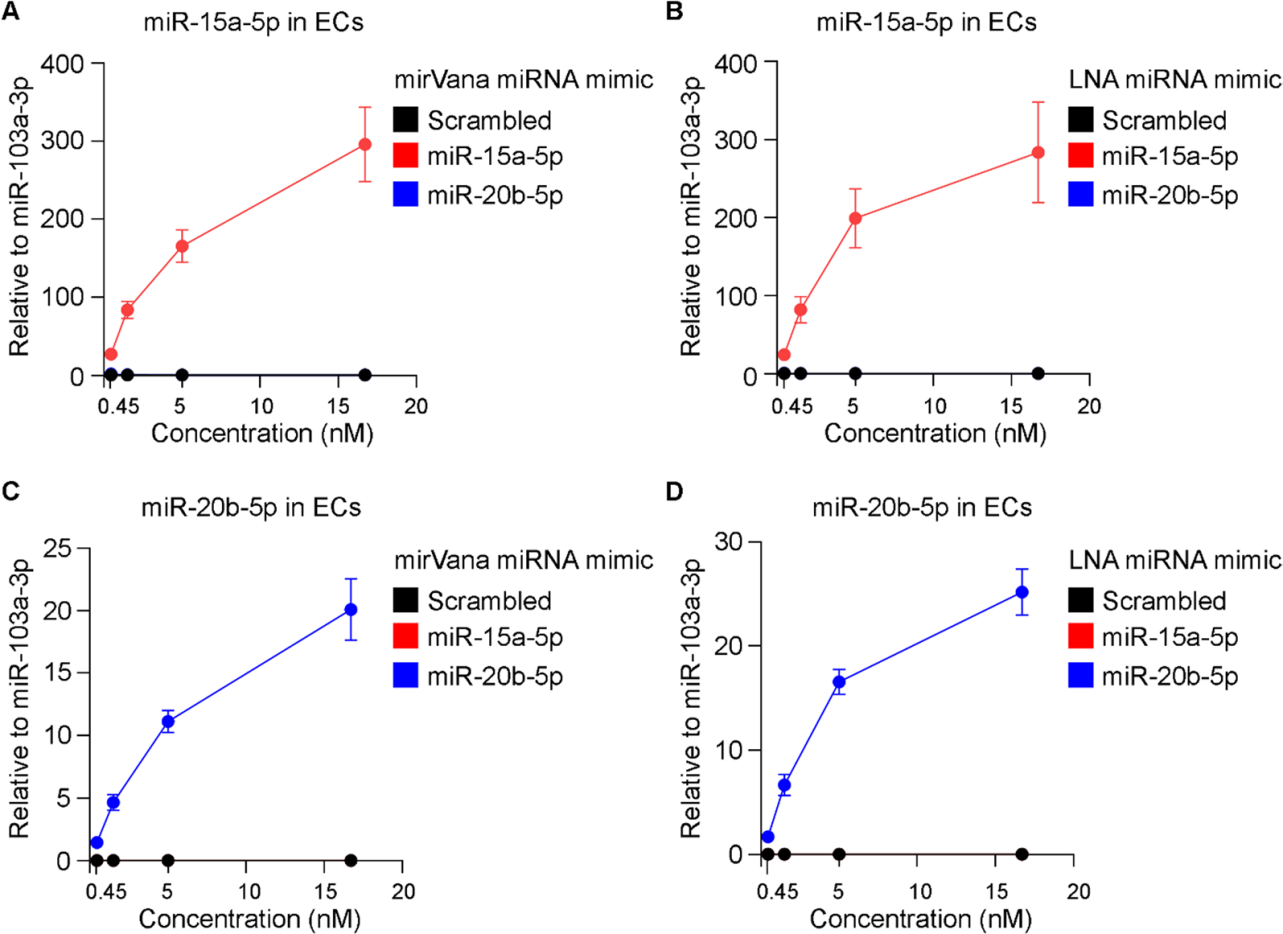
Expression of miR-15a-5p and miR-20b-5p in primary ECs following transfection of the respective miRNA mimics. ECs were transfected with mirVana miRNA mimic for **A** miR-15a-5p and **C** miR-20b-5p or their respective scrambled control at elevated concentration (0.45, 1.5, 5 and 16.7 nM) using Lipofectamine RNAiMAX as a carrier. In addition, ECs were transfected with LNA miRNA mimic for **B** miR-15a-5p and **D** miR-20b-5p or their respective scrambled control at elevated concentration (0.45, 1.5, 5 and 16.7 nM) using Lipofectamine RNAiMAX as a carrier. Following 48 h, total RNAs were isolated and expression levels of miR-15a-5p, miR-20b-5p and reference miRNA miR-103a-3p were measured by qRT-PCR. Relative expression levels of miR-15a-5p and miR-20b-5p in comparison to miR-103a-3p were shown as mean ± SEM. N = 3 experiments and N = 3 technical replicates were performed

As miR-20b-5p is not expressed in ECs, the relative expression of both miR-15a-5p and miR-20b-5p following transfection was determined using miR-103a-3p, which is abundantly expressed in such cells, as a reference. Elevated concentrations of mirVana or LNA miR-15a-5p mimic that were delivered to ECs using Lipofectamine RNAiMAX as a carrier specifically increased the expression of miR-15a-5p (Fig. 4A, B); 0.45 nM of both mirVana and LNA miR-15a-5p mimics resulted in 25-folds increased expression of miR-15a-5p. Nearly 300-folds increase was detected in the expression of miR-15a-5p in ECs when mirVana and LNA mimics were used at 16.7 nM (Fig. 4A, B).

Transfection of ECs with the lowest dose (0.45 nM) of either mirVana or LNA miR-20b-5p mimic resulted in approximately 1.5-folds increased expression of miR-20b-5p. Using 16.7 nM of mirVana or LNA miR-20b-5p mimic resulted in an increased expression of 20–25-folds of miR-20b-5p in the transfected cells (Fig. 4C, D). Importantly, transfection of ECs with mirVana or LNA scrambled control did not affect the expression of either miR-15a-5p or miR-20b-5p. Gross morphological analysis of transfected ECs using a light microscope showed that Lipofectamine RNAiMAX, mirVana miRNA mimics or LNA miRNA mimics didn’t affect cell viability (Additional file 1: Fig. S3A). Flow cytometry analysis of cell surface protein and cell viability validated the expression of CD31 lineage marker and viability of ECs when transfected with Lipofectamine RNAiMAX and 5 nM of mirVana or LNA miRNA mimics (Additional file 1: Fig. S3B).

### Unassisted uptake of miRNA mimics by primary ECs

**Fig. 5:**
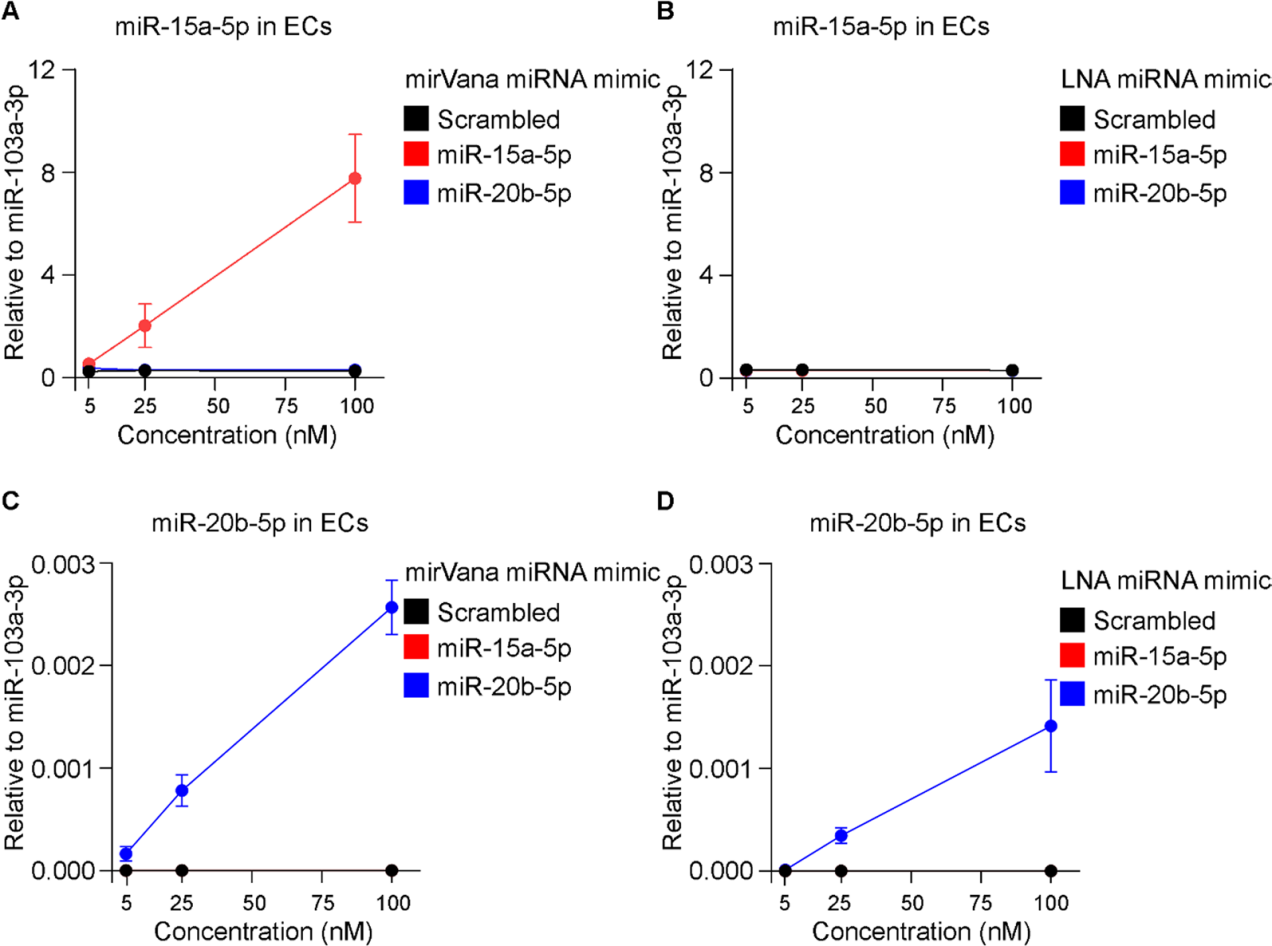
Expression of miR-15a-5p and miR-20b-5p in primary ECs following unassisted uptake of the miRNA mimics. ECs were cultured with elevated concentration (5, 25 and 100 nM) of mirVana miRNA mimic for **A** miR-15a-5p and **C** miR-20b-5p or their respective scrambled control. ECs were also cultured with identical concentration of LNA miRNA mimic for **B** miR-15a-5p and **D** miR-20b-5p, or their respective scrambled control. Following 48 h, total RNAs were isolated and expression levels miR-15a-5p, miR-20b-5p and reference miRNA miR-103a-3p were measured by qRT-PCR. Relative expression levels of miR-15a-5p and miR-20b-5p in comparison to miR-103a-3p were shown as mean ± SEM. N = 2 experiments and N ≥ 2 technical replicates were performed

Next, we tested whether such miRNA mimics can be delivered to ECs without the assistance of a lipid-based carrier. MirVana and LNA miRNA mimics for miR-15a-5p and miR-20b-5p were added to cell culture at 5 nM, 25 nM and 100 nM and the expression of miRNAs was analyzed by qRT-PCR following 48 h. As shown in Fig. 5A, the expression of miR-15a-5p was elevated in ECs following unassisted uptake of the specific mirVana mimic, yet to a lesser extent in comparison to transfection with Lipofectamine RNAiMAX. MiR-20b-5p mimic from mirVana did not affect the expression levels of the respective miRNA (Fig. 5C). Unassisted uptake of LNA miR-15a-5p mimic and miR-20b-5p mimic did not affect the expression levels of miR-15a-5p and miR-20b-5p (Fig. 5B, D).

### Transfection of primary monocytes with miRNA mimics

**Fig. 6:**
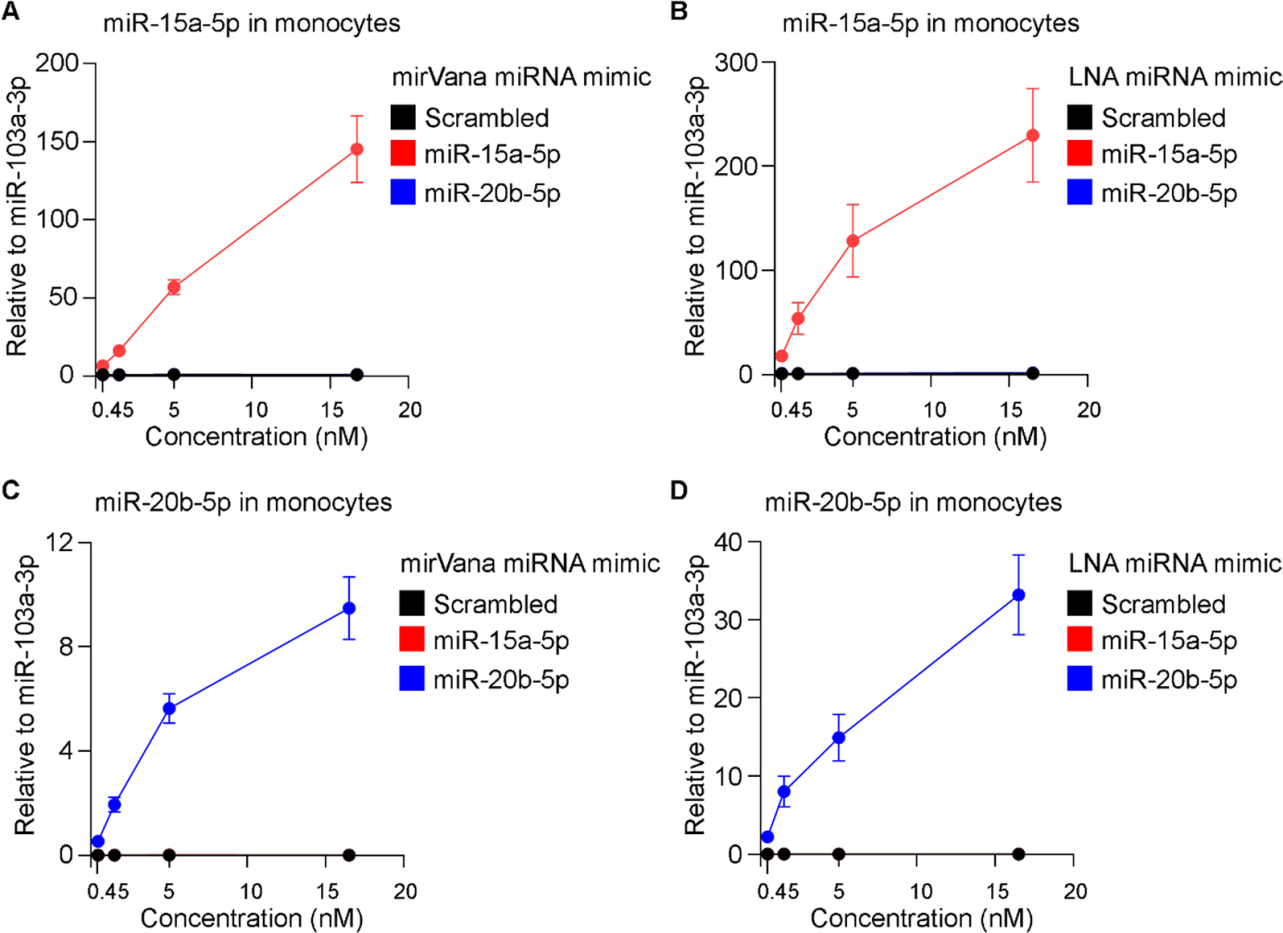
Expression of miR-15a-5p and miR-20b-5p in primary monocytes following transfection of the respective miRNA mimics. Monocytes were transfected with elevated concentration (0.45, 1.5, 5 and 16.7 nM) of mirVana miRNA mimic for **A** miR-15a-5p and **C** miR-20b-5p, or their respective scrambled control using Lipofectamine RNAiMAX as a carrier. In addition, monocytes were transfected with Lipofectamine RNAiMAX in complex with LNA miRNA mimic for **B** miR-15a-5p and **D** miR-20b-5p, or their respective scrambled control at elevated concentration (0.45, 1.5, 5 and 16.7 nM). Following 48 h, total RNAs were isolated and expression levels of miR-15a-5p, miR-20b-5p and reference miRNA miR-103a-3p were measured by qRT-PCR. Relative expression levels of miR-15a-5p and miR-20b-5p in comparison to miR-103a-3p were shown as mean ± SEM. N ≥ 3 experiments and N ≥ 3 technical replicates were performed

As unassisted uptake of miRNA mimics in ECs yielded no effective increase of miRNA expression, we tested the efficiency of transfecting primary monocytes with miR-15a-5p and miR-20b-5p mimics using Lipofectamine RNAiMAX as a carrier. Transfection of primary monocytes with increased concentrations of mirVana or LNA miR-15a-5p mimics upregulated the levels of the respective miRNA in primary monocytes (Fig. 6). Using mirVana or LNA miR-15a-5p mimic at 16.7 nM efficiently increased the expression of miR-15a-5p in 150- and 200-folds, respectively (Fig. 6A, B).

The measured endogenous expression of miR-20b-5p in primary monocytes was 0.0005-fold lower in comparison to miR-103a-3p, which was used as the reference miRNA. Transfection of monocytes with 0.45 nM mirVana or LNA miR-20b-5p mimic resulted in average 0.53–2.21-folds expression of miR-20b-5p in comparison to miR-103a-3p (reference miRNA); this is an effective growth of more than 1000-folds in the levels of miR-20b-5p in the transfected monocytes (Fig. 6C, D). Transfection of mirVana or LNA miR-20b-5p mimic using the highest concentration (16.7 nM) resulted more than 9-folds and 33-folds expression of miR-20b-5p, respectively, relative to miR-103a-3p (Fig. 6C, D). We next assessed effects of transfection on monocyte lineage markers and cell viability. Analysis of cell surface proteins on monocytes transfected with Lipofectamine RNAiMAX and miRNA mimics showed that monocytes express similar levels CD14 and CD63 lineage markers 48 h after transfection with 5 nM of mirVana or LNA miRNA mimics (Additional file 1: Fig. S4A). Flow cytometry analysis of cell viability showed no toxicity on monocytes when transfected with Lipofectamine RNAiMAX and 5 nM of mirVana or LNA miRNA mimics (Additional file 1: Fig. S4B). Taken together, these results showed that delivery of miRNA mimics with mirVana and LNA chemistry are both efficient to increase miRNA levels in ECs and primary monocytes when delivered with lipid preparations.

## Discussion

In this study, we investigated performance of commercially available miRNA inhibitors and mimics in human primary cells, with or without the use of a lipid-based carrier. Three different chemically modified miRNA inhibitors of miR-15a-5p, and two distinct miR-15a-5p and miR-20b-5p mimics were transfected into primary ECs, which were used as a model for proliferating adherent cells. In addition, the performance of the miRNA inhibitors and mimics was further validated in primary human monocytes, being non-proliferating and non-adherent cells. Our findings suggest that LNA miRNA inhibitors are superior when delivered with a lipid-based carrier. More specifically, LNA-PS miRNA inhibitor with chemically modified nucleotide bonds can be used without a lipid-based carrier to effectively knock down miRNA expression in human primary cells. Delivery of miRNA mimics to primary ECs and monocytes was efficiently achieved using either mirVana or LNA chemistry when performed with lipid preparations.

MiRNAs are essential for post-transcriptional gene regulation and subsequent cell function, and their dysregulation is associated with various diseases [3–5]. Downregulation or overexpression of specific miRNAs in vitro, or in a living organism, are essential to study the role of miRNAs in physiology and pathology. Several studies explored the effects of specific miRNAs using miRNA inhibitors and mimics with different oligonucleotide chemistries and doses. Most of these studies, however, were performed in cell lines and used lipid-based carriers [23–25]. It is therefore unclear whether lipid-based carriers are essential to deliver miRNA inhibitors and mimics to cells. Consequently, data regarding the concentration of miRNA inhibitors and mimics that can be used in human primary cells are lacking. Therefore, we tested three different miRNA inhibitors and two different miRNA mimics in primary human ECs and monocytes using two different delivery methods.

Deletion or silencing of miR-15a/16-1 cluster is found in several blood disorders in humans [26–28]. Deletion of miR-15a/16-1 cluster results in blood disorders in mice confirming the causative role of miR-15a/16-1 in human diseases and highlighting the role of miRNA cluster in functional regulation of blood cells [29–31]. The physiological role of miR-15a/16-1 is, however, tissue-specific as endothelium-specific deletion of miR-15a/16-1 cluster improves brain function and blood–brain barrier structure after brain injury [21, 22]. These findings suggest that gain- and loss- of function of miR-15a used as a therapeutic target in a tissue-specific manner. In contrast to miR-15a, the expression level of miR-20b is low in healthy cells [32]. An increased expression of miR-20b was shown in tissue samples from several different types of cancers [33, 34].

In this work we have explored in detail the concentration that are needed to effectively achieve downregulation or overexpression of specific miRNAs. We showed that the effectiveness of miR-15a-5p inhibitors to downregulate the target miRNA levels varies with different chemistries. Our analysis shows that LNA miRNA inhibitors effectively knocked down their target miRNA when used at 30 nM following 24 h. In comparison, mirVana miRNA inhibitor modestly downregulated miR-15a-5p levels, even when administrated at a concentration as high as 100 nM, neither following longer incubation, nor using two consecutive transfections. In line with our findings, variable inhibition of miRNA levels was achieved using mirVana miRNA inhibitors [12]. The authors delivered miRNA inhibitors against miR-424-5p, miR-29a-3p and miR-16-5p to ECs with Lipofectamine RNAiMAX and used 90 nM of miRNA inhibitors which is close to the highest dose (100 nM) we tested in transfection experiments. Their results showed significant downregulation of all tested miRNAs, though they only achieved 50% downregulation of one of the miRNAs. MirVana miRNA inhibitors were recently used in a different study at 10 nM to silence miRNA expression in HEK293 cell line, where the targeted miRNA levels were analyzed at 48 h after transfection [24]. Another study reports transfection of HEK293T and DAOY cell lines with mirVana miRNA inhibitors using 200 pmol and analysis at 72 h after transfection [23]. While levels of target miRNAs after transfection experiments were not reported in these studies, it is possible that silencing with certain miRNA inhibitors are more efficient in cell lines compared to primary cell cultures.

Efficiency, safety, and cost-effectiveness should be considered before determining the most suitable strategy for inhibition or overexpression of miRNAs in functional studies or therapeutic approaches. Chemically modified synthetic miRNA inhibitors and mimics are among the approaches that were developed to control miRNA expression. While high production costs might limit their use [35], synthetic miRNA inhibitors and mimics are readily available to use in experimental settings. Transfection conditions and concentrations, however, may need to be adjusted according to the target cellular system. Vector-based systems to inhibit or overexpress target miRNAs were developed as alternative approaches to synthetic oligonucleotides [36, 37]. MiRNA sponges, which are plasmid DNA-encoded miRNA inhibitors, contain multiple tandem miRNA binding sites to competitively inhibit miRNA function [37, 38]. Alternatively, miRNA or anti-miRNA sequences can be inserted into plasmid vectors and delivered to cells to modify miRNA expression. Such vector-based miRNA inhibitors or mimics can provide efficient, sustained, and inducible expression in controlling target miRNAs. However, efficient delivery and strong expression of plasmid DNA vectors in primary cells and tissues are usually achieved by inserting these vectors into viral expression systems [25, 38]. Production of high-titer virus particles and maintenance of a cell culture laboratory in accordance with biosafety regulations might decrease cost-effectiveness of this strategy. Moreover, random genetic integration of viral vectors, e.g., lentiviral expression systems, may result in insertional mutagenesis in target cells.

Cellular delivery strategy is one of the most important factors affecting clinical success of oligonucleotide therapeutics. One of these delivery methods is intracellular delivery of antisense oligonucleotides in the absence of a lipid-based carrier or molecular conjugation to silence gene expression (39). Oligonucleotides were modified chemically (e.g., LNA substitution and phosphorothioate modification) to increase their nuclease resistance and gene silencing efficiency [40]. This method, which is termed gymnosis, was shown as an efficient way to silence gene expression in cell lines using antisense oligonucleotides [39, 40]. Nevertheless, the efficiency of gymnosis in delivery of miRNA inhibitors or mimics to human primary cells has not been reported previously. Here, we compared the performance of LNA miR-15a-5p inhibitors with two different chemical structures in the absence of a lipid-based carrier. LNA-PS miRNA inhibitor efficiently downregulated the endogenous levels of miR-15a-5p, achieving over 95% downregulation when used at 100 nM in ECs and 30 nM in primary monocytes. Such unassisted delivery of an LNA miR-155 inhibitor was previously tested in low-grade lymphoma cell lines [41]. The authors used a high concentration of the inhibitor (25 µM) in comparison to concentrations that were examined in this work to attain an efficient inhibition of the respective miRNA. It should be noted, however, that the authors used an LNA-PE miRNA inhibitor, which we found to be less effective. In our hands, 600 nM of LNA-PE inhibitor showed a modest efficiency when delivered to primary ECs and monocytes without the assistance of a lipid-based carrier. It is important to note that although unassisted delivery of LNA-PS miR-15a-5p inhibitor to both ECs and monocyte was highly efficient, it may require longer transfection time, as gymnosis requires physiological uptake of the oligonucleotides by the cells.

The reported efficiency of transfecting cells with miRNA mimics varies and can be affected by several factors, with the main ones being the reagents and concentrations that were used, and the harvesting time. The transfection reagents are commonly used for intracellular delivery of oligonucleotides and can be source of cellular toxicity. Screening of commercially available transfection reagents showed that Lipofectamine RNAiMAX is a better option for the majority of cells due to its low cytotoxicity [42]. Lipofectamine RNAiMAX was also used in this study. Indeed, microscopic and flow cytometry analysis of cell viability and surface markers showed that Lipofectamine RNAiMAX had no harmful effects on primary cells.

Although functional dissection of the role of miRNA following upregulation using miRNA mimics can be performed in cell lines [23–25], studies of human primary cells are more relevant to delineate physiological roles of miRNAs. However, little work has been performed using miRNA mimics in human primary cells. In one study using, LNA miR-199a-5p mimic was transfected to primary ECs [43]. The authors used 20 nM of LNA miR-199a-5p mimic and achieved 60-folds overexpression of the miRNA 28 h after transfection. In another study, primary human hepatocytes were transfected with 25 nM of miR-125b-5p mimic [44]. In this work, we achieved a similar level of overexpression using 1.5 nM of either mirVana or LNA miR-15a-5p mimic when delivered with a lipid-based carrier. Unlike miRNA inhibitors, unassisted delivery of miRNA mimics was found to be inefficient. One reason that prevents uptake of miRNA mimics by the cells without lipid-based carriers may be their chemical structure. While miRNA inhibitors are single-stranded oligonucleotides, miRNA mimics are synthesized as double or triple stranded oligonucleotides to enhance their cellular processing as endogenous miRNAs. Another factor that facilitates the gymnotic uptake of particularly LNA-PS miRNA inhibitors is the phosphorothioate internucleotide bonds, making the miRNA inhibitor more resistant to nucleases. MiRNA mimics include LNA-enhanced passenger strands to increase their efficiency though the strands contain natural phosphodiester bonds and are not further enhanced by phosphorothioate bonds.

In this study, we evaluated kinetics of three different miRNA inhibitors of miR-15a-5p and two different mimics of miR-15a-5p and miR-20b-5p in primary human ECs and monocytes. Our results provide an optimized workflow to achieve efficient knockdown or overexpression of miRNAs in human primary cells and can be a valuable resource for pharmacokinetic studies targeting miRNAs and their role in human diseases.

## Supplementary Information

**Additional file 1. Supplementary Figure 1.** Analysis of primary ECs by flow cytometry following one-time or double transfection with miRNA inhibitors. **Supplementary Figure 2.** Analysis of monocyte purity by flow cytometry following enrichment from PBMCs using negative selection. **Supplementary Figure 3.** Analysis of primary ECs by light microscopy and flow cytometry following transfection with the respective miRNA mimics. **Supplementary Figure 4.** Analysis of monocytes by flow cytometry following transfection with the respective miRNA mimics.

## Data Availability

All data generated or analyzed during this study are included in this article and its supplementary information files.

## References

[1] Bartel DP (2018). Metazoan MicroRNAs. Cell.

[2] Mehta A, Baltimore D (2016). MicroRNAs as regulatory elements in immune system logic. Nat Rev Immunol.

[3] Peters LJF, Biessen EAL, Hohl M, Weber C, van der Vorst EPC, Santovito D (2020). Small things matter: relevance of microRNAs in cardiovascular disease. Front Physiol.

[4] Lin S, Gregory RI (2015). MicroRNA biogenesis pathways in cancer. Nat Rev Cancer.

[5] Abe M, Bonini NM (2013). MicroRNAs and neurodegeneration: role and impact. Trends Cell Biol.

[6] Rupaimoole R, Slack FJ (2017). MicroRNA therapeutics: towards a new era for the management of cancer and other diseases. Nat Rev Drug Discov.

[7] Khvorova A, Watts JK (2017). The chemical evolution of oligonucleotide therapies of clinical utility. Nat Biotechnol.

[8] Cardarelli F, Digiacomo L, Marchini C, Amici A, Salomone F, Fiume G (2016). The intracellular trafficking mechanism of Lipofectamine-based transfection reagents and its implication for gene delivery. Sci Rep.

[9] Samaridou E, Heyes J, Lutwyche P (2020). Lipid nanoparticles for nucleic acid delivery: current perspectives. Adv Drug Deliv Rev.

[10] Tousignant JD, Gates AL, Ingram LA, Johnson CL, Nietupski JB, Cheng SH (2000). Comprehensive analysis of the acute toxicities induced by systemic administration of cationic lipid: plasmid DNA complexes in mice. Hum Gene Ther.

[11] van Rooij E, Olson EN (2012). MicroRNA therapeutics for cardiovascular disease: opportunities and obstacles. Nat Rev Drug Discov.

[12] Rosano S, Corà D, Parab S, Zaffuto S, Isella C, Porporato R (2020). A regulatory microRNA network controls endothelial cell phenotypic switch during sprouting angiogenesis. Elife.

[13] Martello A, Mellis D, Meloni M, Howarth A, Ebner D, Caporali A (2018). Phenotypic miRNA screen identifies miR-26b to promote the growth and survival of endothelial cells. Mol Ther Nucleic Acids.

[14] Suárez Y, Wang C, Manes TD, Pober JS (2010). Cutting edge: TNF-induced microRNAs regulate TNF-induced expression of e-selectin and intercellular adhesion molecule-1 on human endothelial cells: feedback control of inflammation. J Immunol.

[15] Bridge G, Monteiro R, Henderson S, Emuss V, Lagos D, Georgopoulou D (2012). The microRNA-30 family targets DLL4 to modulate endothelial cell behavior during angiogenesis. Blood.

[16] Kim S, Lee K-S, Choi S, Kim J, Lee D-K, Park M (2018). NF-kB–responsive miRNA-31-5p elicits endothelial dysfunction associated with preeclampsia via down-regulation of endothelial nitric-oxide synthase. J Biol Chem.

[17] Naqvi AR, Fordham JB, Nares S (2015). miR-24, miR-30b, and miR-142-3p regulate phagocytosis in myeloid inflammatory cells. J Immunol.

[18] Widlansky ME, Jensen DM, Wang J, Liu Y, Geurts AM, Kriegel AJ (2018). miR-29 contributes to normal endothelial function and can restore it in cardiometabolic disorders. EMBO Mol Med.

[19] Peltier HJ, Latham GJ (2008). Normalization of microRNA expression levels in quantitative RT-PCR assays: identification of suitable reference RNA targets in normal and cancerous human solid tissues. RNA.

[20] Bargaje R, Hariharan M, Scaria V, Pillai B (2010). Consensus miRNA expression profiles derived from interplatform normalization of microarray data. RNA.

[21] Ma F, Sun P, Zhang X, Hamblin MH, Yin K-J (2020). Endothelium-targeted deletion of the miR-15a/16-1 cluster ameliorates blood-brain barrier dysfunction in ischemic stroke. Sci Signal.

[22] Sun P, Zhang K, Hassan SH, Zhang X, Tang X, Pu H (2020). Endothelium-targeted deletion of microRNA-15a/16-1 promotes poststroke angiogenesis and improves long-term neurological recovery. Circ Res.

[23] Nitschke L, Tewari A, Coffin SL, Xhako E, Pang K, Gennarino VA (2020). miR760 regulates ATXN1 levels via interaction with its 5′ untranslated region. Genes Dev.

[24] Yasukawa K, Kinoshita D, Yaku K, Nakagawa T, Koshiba T (2020). The microRNAs miR-302b and miR-372 regulate mitochondrial metabolism via the SLC25A12 transporter, which controls MAVS-mediated antiviral innate immunity. J Biol Chem.

[25] Lee YH, Jang H-J, Kim S, Choi SS, Khim KW, Eom H-J (2021). Hepatic MIR20B promotes nonalcoholic fatty liver disease by suppressing PPARA. Elife.

[26] Calin GA, Dumitru CD, Shimizu M, Bichi R, Zupo S, Noch E (2002). Frequent deletions and down-regulation of micro-RNA genes *miR15* and *miR16* at 13q14 in chronic lymphocytic leukemia. Proc Natl Acad Sci.

[27] Lovat F, Nigita G, Distefano R, Nakamura T, Gasparini P, Tomasello L (2020). Combined loss of function of two different loci of miR-15/16 drives the pathogenesis of acute myeloid leukemia. Proc Natl Acad Sci.

[28] Calin GA, Ferracin M, Cimmino A, Di Leva G, Shimizu M, Wojcik SE (2005). A microRNA signature associated with prognosis and progression in chronic lymphocytic leukemia. N Engl J Med.

[29] Klein U, Lia M, Crespo M, Siegel R, Shen Q, Mo T (2010). The DLEU2/miR-15a/16-1 cluster controls B cell proliferation and its deletion leads to chronic lymphocytic leukemia. Cancer Cell.

[30] Gagnon JD, Kageyama R, Shehata HM, Fassett MS, Mar DJ, Wigton EJ (2019). miR-15/16 restrain memory T cell differentiation, cell cycle, and survival. Cell Rep.

[31] Sewastianik T, Straubhaar JR, Zhao J-J, Samur MK, Adler K, Tanton HE (2021). miR-15a/16-1 deletion in activated B cells promotes plasma cell and mature B-cell neoplasms. Blood.

[32] Rose SA, Wroblewska A, Dhainaut M, Yoshida H, Shaffer JM, Bektesevic A (2021). A microRNA expression and regulatory element activity atlas of the mouse immune system. Nat Immunol.

[33] Huang T, Alvarez AA, Pangeni RP, Horbinski MC, Lu S, Kim S-H (2016). A regulatory circuit of miR-125b/miR-20b and Wnt signalling controls glioblastoma phenotypes through FZD6-modulated pathways. Nat Commun.

[34] Yu J, Chen S, Niu Y, Liu M, Zhang J, Yang Z (2020). Functional significance and therapeutic potential of miRNA-20b-5p in esophageal squamous cell carcinoma. Mol Ther Nucleic Acids.

[35] Bak RO, Hollensen AK, Mikkelsen JG (2013). Managing microRNAs with vector-encoded decoy-type inhibitors. Mol Ther.

[36] Winkle M, El-Daly SM, Fabbri M, Calin GA (2021). Noncoding RNA therapeutics—challenges and potential solutions. Nat Rev Drug Discov.

[37] Ebert MS, Neilson JR, Sharp PA (2007). MicroRNA sponges: competitive inhibitors of small RNAs in mammalian cells. Nat Methods.

[38] Xie J, Ameres SL, Friedline R, Hung J-H, Zhang Y, Xie Q (2012). Long-term, efficient inhibition of microRNA function in mice using rAAV vectors. Nat Methods.

[39] Stein CA, Hansen JB, Lai J, Wu S, Voskresenskiy A, H⊘g A (2009). Efficient gene silencing by delivery of locked nucleic acid antisense oligonucleotides, unassisted by transfection reagents. Nucleic Acids Res.

[40] Souleimanian N, Deleavey GF, Soifer H, Wang S, Tiemann K, Damha MJ, et al. Antisense 2′-Deoxy, 2′-Fluoroarabino Nucleic Acid (2′F-ANA) Oligonucleotides: in vitro gymnotic silencers of gene expression whose potency is enhanced by fatty acids. Mol Ther Nucleic Acids. 2012;1:e43. 10.1038/mtna.2012.35 PMC3499694 23344235

[41] Zhang Y, Roccaro AM, Rombaoa C, Flores L, Obad S, Fernandes SM (2012). LNA-mediated anti-miR-155 silencing in low-grade B-cell lymphomas. Blood.

[42] Wang T, Larcher LM, Ma L, Veedu RN (2018). Systematic screening of commonly used commercial transfection reagents towards efficient transfection of single-stranded oligonucleotides. Molecules.

[43] Heuslein JL, Gorick CM, McDonnell SP, Song J, Annex BH, Price RJ (2018). Exposure of endothelium to biomimetic flow waveforms yields identification of miR-199a-5p as a potent regulator of arteriogenesis. Mol Ther Nucleic Acids.

[44] Yang D, Yuan Q, Balakrishnan A, Bantel H, Klusmann J-H, Manns MP (2016). MicroRNA-125b-5p mimic inhibits acute liver failure. Nat Commun.

