# Supplementary material for: Optimized workflow to modify microRNA expression in primary human intravascular cells
Source: BMC Immunol. 2023 Feb 15;24:5. doi: 10.1186/s12865-023-00540-9 (PMC9933393; doi:10.1186/s12865-023-00540-9)
Supplement: Supplementary file 1 — Additional file 1. Supplementary Figure 1. Analysis of primary ECs by flow cytometry following one-time or double transfection with miRNA inhibitors. Supplementary Figure 2. Analysis of monocyte purity by flow cytometry following enrichment from PBMCs using negative selection. Supplementary Figure 3. Analysis of primary ECs by light microscopy and flow cytometry following transfection with the respective miRNA mimics. Supplementary Figure 4. Analysis of monocytes by flow cytometry following transfection with the respective miRNA mimics. [file 12865_2023_540_MOESM1_ESM.docx]

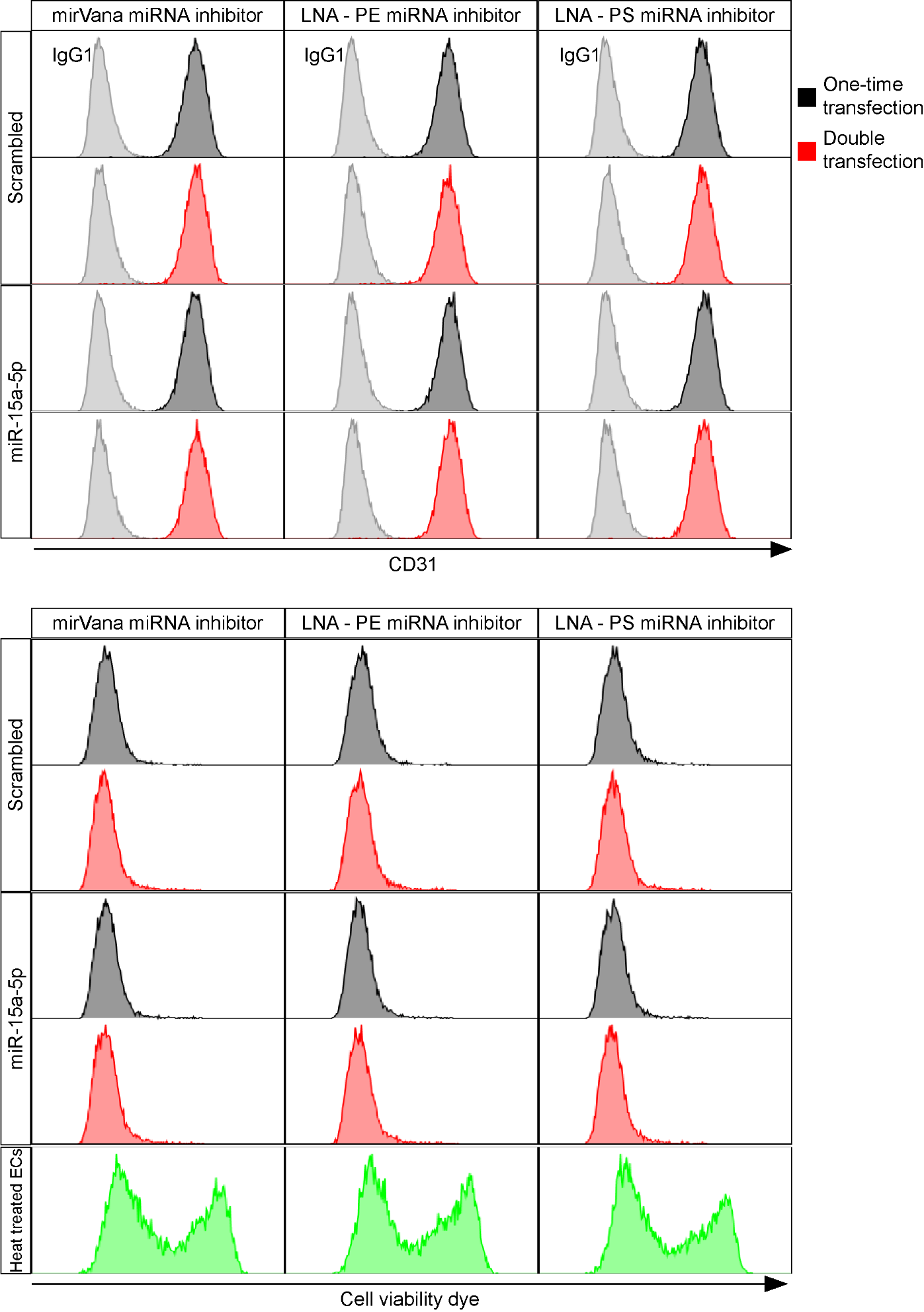


**Supplementary Figure 1.** Analysis of primary ECs by flow cytometry following one-time or double transfection with miRNA inhibitors. ECs were transfected with 30 nM of mirVana, LNA-PE, or LNA-PS miR-15a-5p miRNA inhibitor or their respective scrambled control in complex with Lipofectamine RNAiMAX. Following 24 hours, cell culture media was replaced with a freshly prepared EC media. Cells were either kept in fresh EC media for additional 24 hours (one-time transfection) or transfected for the second time (double transfection) with the respective miRNA inhibitors (30 nM) in complex with Lipofectamine RNAiMAX. Expression of EC lineage marker CD31 (upper panel) and cell viability (lower panel) was analyzed 48 hours after the first transfection (24 hours after the second transfection). Heat treated ECs were used as positive control for staining to separate live cells (left) and dead cells (right) on the histogram. N = 2 experiments (in total N > 9000 ECs) were concatenated.


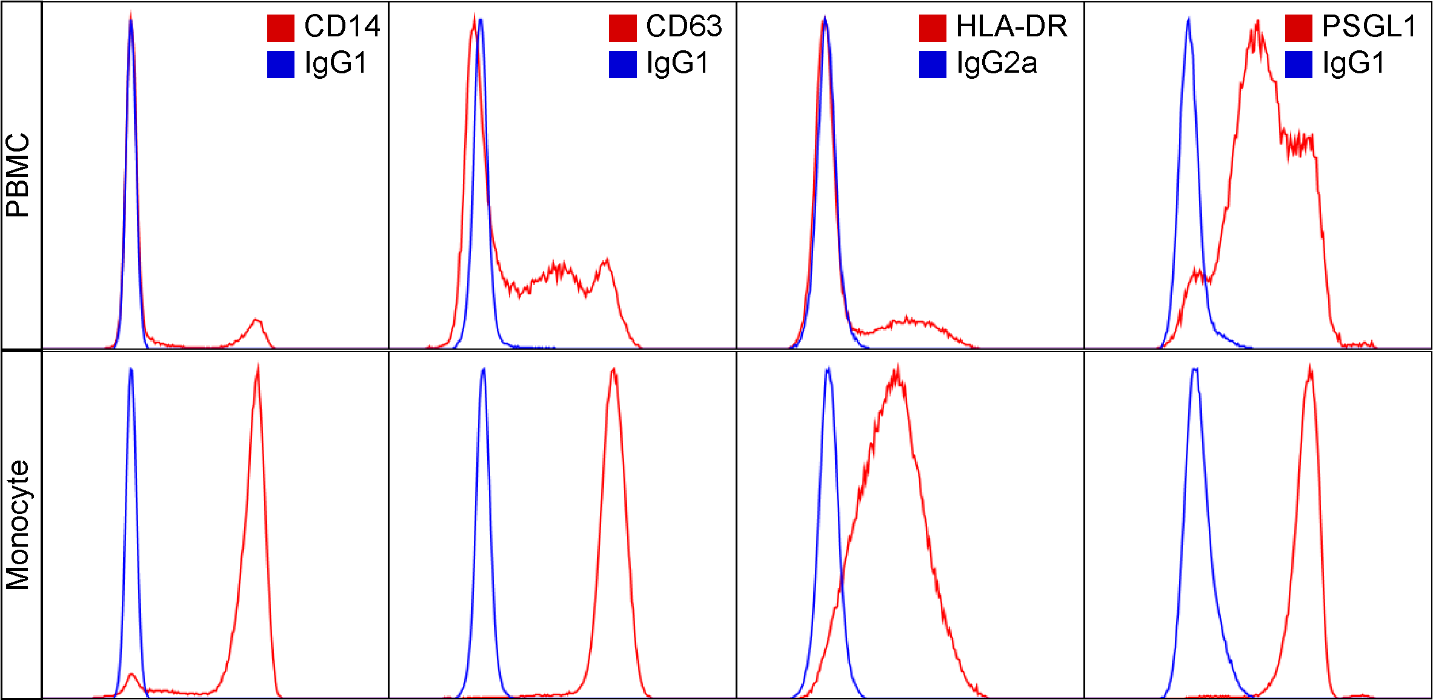


**Supplementary Figure 2.** Analysis of monocyte purity by flow cytometry following enrichment from PBMCs using negative selection. The expression of monocyte lineage markers was analyzed on PBMCs before monocyte isolation (upper panel) and on negatively selected monocytes (lower panel). N = 6 experiments (in total N > 65000 PBMCs and N > 125000 monocytes) were concatenated.


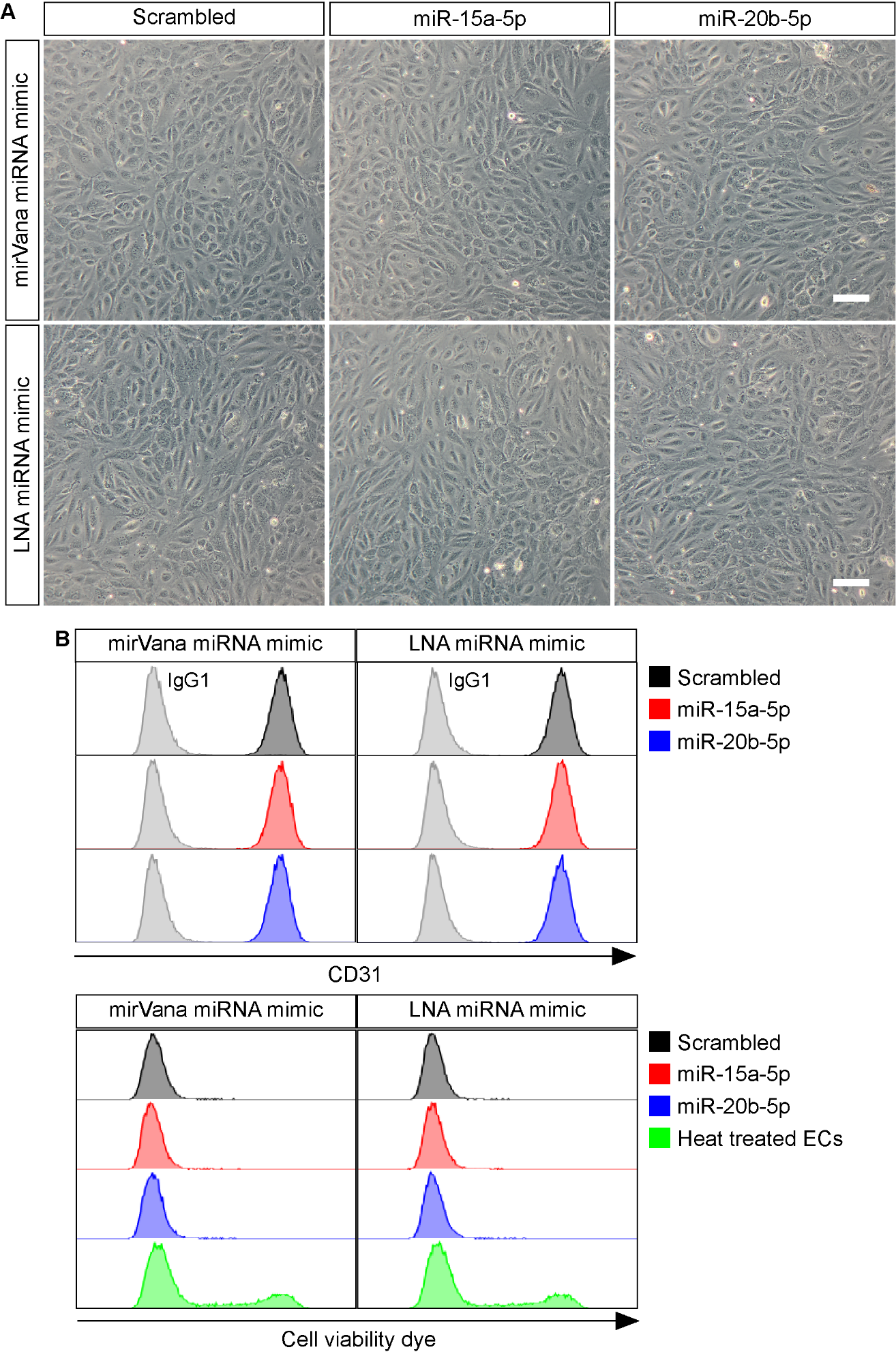


**Supplementary Figure 3.** Analysis of primary ECs by light microscopy and flow cytometry following transfection with the respective miRNA mimics. **(A)** Phase-contrast images of live ECs were taken 48 hours after transfection with mirVana or LNA miRNA mimic (5 nM) complexed with Lipofectamine RNAiMAX. Representative images are shown from N = 2 experiments. Scale bar represents 100 μM. **(B)** Expression of EC lineage marker CD31 (upper panel) and cell viability (lower panel) was analyzed 48 hours after transfection with mirVana or LNA miRNA mimic (5 nM) complexed with Lipofectamine RNAiMAX. Heat treated ECs were used as positive control for staining to separate live cells (left) and dead cells (right) on the histogram. N = 2 experiments (in total N > 13000 ECs) were concatenated.


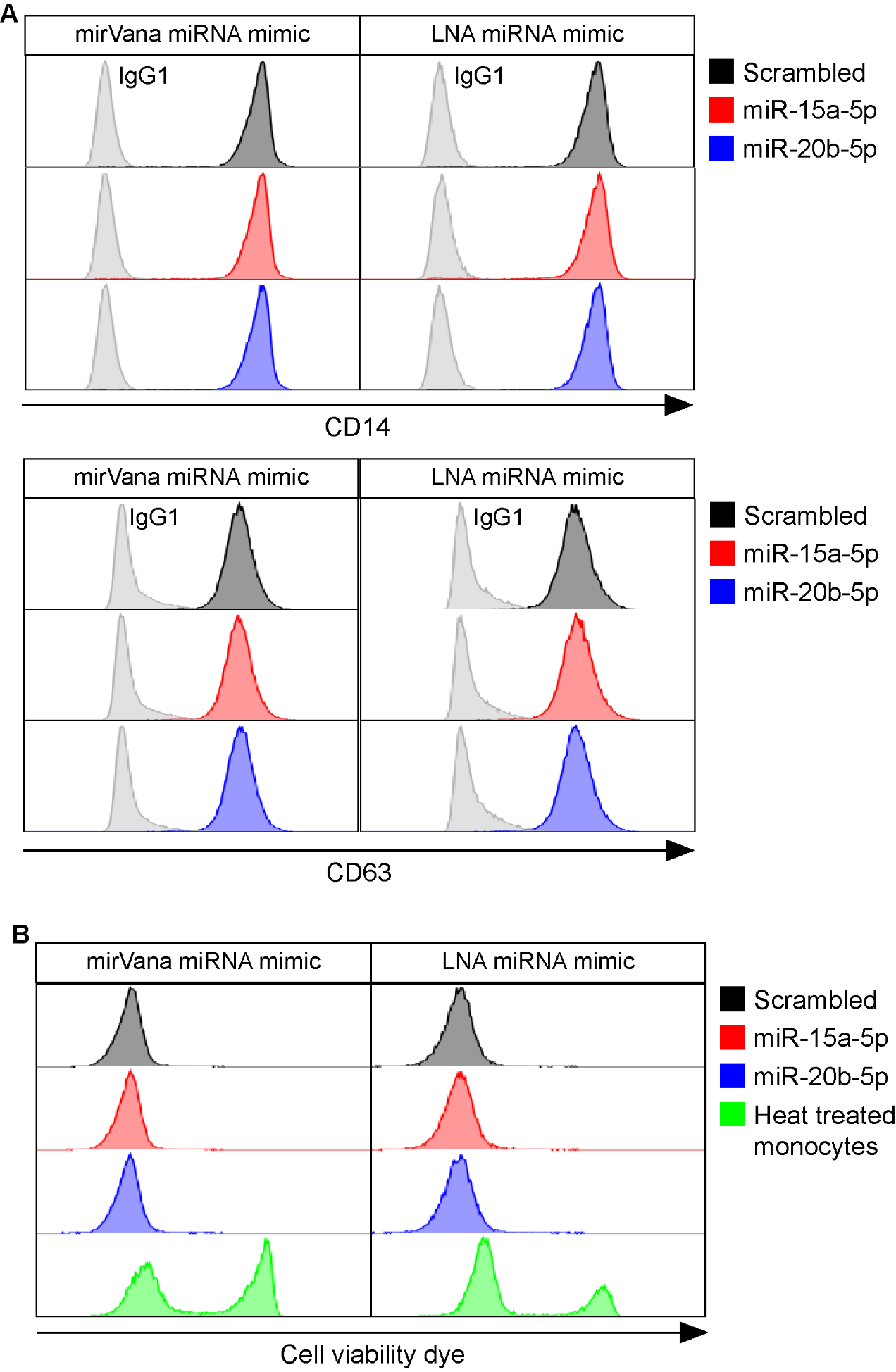


**Supplementary Figure 4.** Analysis of monocytes by flow cytometry following transfection with the respective miRNA mimics. **(A)** Expression of monocyte lineage markers was analyzed on monocytes 48 hours after transfection with mirVana or LNA miRNA mimic (5 nM) complexed with Lipofectamine RNAiMAX. N = 2 experiments (N > 30000 monocytes) were concatenated. **(B)** Cell viability of monocytes was assessed 48 hours after transfection with mirVana or LNA miRNA mimic (5 nM) complexed with Lipofectamine RNAiMAX. Heat treated monocytes were used as positive control for staining to separate live cells (left) and dead cells (right) on the histogram. N = 2 experiments (in total N > 30000 monocytes) were concatenated.
